# From here…… where and how?

**DOI:** 10.4103/0972-0707.57630

**Published:** 2009

**Authors:** Velayutham Gopikrishna

**Affiliations:** Editor-in-Chief, Journal of Conservative Dentistry, Department of Conservative Dentistry, Meenakshi Ammal Dental College, Alapakkam Main Road, Maduravoyal, Chennai – 600 095, Tamil Nadu, India. E-mail: editor@jcd.org.in

The growth of our specialty of Endodontics in the last few decades has been tremendous in terms of reach and impact of improving the overall quality of general oral health. The greatest contribution of our field to dentistry has been the change in perception of patients towards dentistry舰from a profession synonymous with extractions and pain to a profession which specializes in saving and conserving teeth. However, the biggest unanswered challenges our specialty faces are:

- The fear of pain attributed to root canal therapy amongst patients
  Fear of the dentist plagues more than 80% adults; and more than half say fear may keep them from going to see the dentist, according to a new survey by the American Association of Endodontists (AAE). While fear of pain is the top reason adults avoid the dentist, root canal treatment is the *most feared dental procedure*, according to the AAE survey. In fact, adults are as afraid of getting a root canal (54%) as they are of flying on an airplane during a storm (57%) and are more fearful of the procedure than of speaking in public or interviewing for a job (both at 42%).
  Nearly one-third of adult respondents admitted that their fear of the dentist is based on hearing about someone else′s experience rather than their own. An ironic situation, since another survey showed most people who have had root canal treatment performed by a specialist report it actually was a positive experience!
- Lack of awareness amongst patients regarding our specialty
  There is lack of awareness amongst the public that specialist dental care exists in their community. However, most of the times when we introduce ourselves as endodontists; we either get a reply saying *“Endo…. who?!!”* or a blank stare even amongst medical doctors and specialists. This is where the role of our society is important in educating and propagating the merits and skills of our specialty.
  There is a universal trend across the world for general practitioners to practice endodontics. This cannot be prevented or prohibited. Prudence dictates that the best way forward for our specialty is to educate the consume…the general public about who we are….

This can be done as a collective effort in the form of the following initiatives:

- **Retreatodontics:** The future of endodontists would be partly focused on retreatment of failed root canals, which is a viable alternative to more invasive and expensive alternatives such as implants.
- Have an annual **Root Canal Awareness Week**, similar to the diabetes or the heart care campaign, which would be simultaneously held across the country educating general dentists and public about our capabilities and newer concepts of treatment.
- **Use the Media as an effective partner** by periodically giving press releases of the position of FODI and IES in relation to key endodontic related practices and principles. Eg: The view of FODI and IES on the endodontics vs. implants debate….
- **Practitioners as Partners:** We should view general practitioners as our partners and establish a healthy professional relationship. Educating the practitioners through the CDE meets that there are cases which need specialist attention and equipments would lead to a healthy trend of “referrals”.
- **Educate the Patient:** Using the FODI and IES webpage to act as a source of information to the patient in answering their queries and fears would be the best way to connect to them.
- Use the FODI and IES website to create **“Your Friendly Neighborhood Endodontist”** page which would give the database for patients to find the specialists certified by our society across the country.

It is said that there is nothing permanent but change…. It is time for us to change and be more pro-active in transforming the face of Endodontic practice in our country.

Be the change you wish to see!!!

Food for thought…..



